# Comparative outcomes of pars plana vitrectomy with subretinal rt-PA injection in polypoidal choroidal vasculopathy: the role of simultaneous anti-VEGF treatment

**DOI:** 10.3389/fmed.2025.1565507

**Published:** 2025-07-03

**Authors:** Lulu Chen, Chuting Wang, Wenfei Zhang, Lihui Meng, Lu Sun, Youxin Chen

**Affiliations:** ^1^Department of Ophthalmology, Peking Union Medical College Hospital, Chinese Academy of Medical Sciences, Beijing, China; ^2^Key Laboratory of Ocular Fundus Diseases, Chinese Academy of Medical Sciences & Peking Union Medical College Hospital, Beijing, China

**Keywords:** submacular hemorrhage, polypoidal choroidal vasculopathy, recombinant tissue plasminogen activator, simultaneous anti-VEGF, surgical outcome

## Abstract

**Purpose:**

This study aimed to assess the efficacy of simultaneous intravitreal anti-vascular endothelial growth factor (VEGF) with pars plana vitrectomy (PPV), subretinal recombinant tissue plasminogen activator (rt-PA), and pneumatic tamponade in treating submacular hemorrhage (SMH) due to polypoidal choroidal vasculopathy (PCV), compared to the procedure without anti-VEGF.

**Methods:**

We retrospectively analyzed PCV patients with SMH who underwent the procedure at a tertiary hospital from 2021 to 2024. Outcomes comprised alterations in best-corrected visual acuity (BCVA), SMH absorption, and the administration of postoperative anti-VEGF injections.

**Results:**

A total of 31 patients were included in the study. There was no significant difference in best-corrected visual acuity (BCVA) improvement or anti-VEGF usage between the anti-VEGF group and the control group. Both treatment strategies led to improved visual acuity. The mean number of anti-VEGF injections following surgery was 1.0 (8.0) in the anti-VEGF group and 1.0 (6.0) in the control group. Complications such as retinal detachment and recurrent vitreous hemorrhage occurred in both groups, with no significant difference between the groups.

**Conclusion:**

Simultaneous intravitreal anti-VEGF did not outperform the procedure without it in terms of improved visual acuity or postoperative anti-VEGF usage. Further studies are needed to determine the best treatment approach for PCV patients with SMH.

## Introduction

Submacular hemorrhage (SMH) is an acute and severe vision-threatening complication associated with polypoidal choroidal vasculopathy (PCV). This condition is defined by the accumulation of blood in the subretinal space, which is located between the neurosensory retina and the retinal pigment epithelium (RPE). Such accumulation can significantly disrupt the retinal structure and impair visual function. Patients with SMH may experience a sudden loss of vision, necessitating timely treatment, as subretinal hemorrhage can lead to damage to the outer retinal layers, resulting in irreversible vision loss (1).

PCV is a subtype of age-related macular degeneration (AMD) with a higher prevalence among Asian patients. It is characterized by a branching neovascular network and polypoidal lesions. The incidence of massive SMH has been reported to be 2.45% within 1 year of initial diagnosis, increasing to 29.85% within 10 years of the initial visit (2). Treatment options for SMH secondary to PCV include intravitreal anti-vascular endothelial growth factor (anti-VEGF), intravitreal recombinant tissue plasminogen activator (rt-PA), gas injection, subretinal rt-PA in combination with pars plana vitrectomy (PPV) and gas tamponade, and others (3–5). rt-PA dissolves hemorrhages by breaking down fibrin and coagulation factors, while gas tamponade aids in displacing subretinal blood. Subretinal injection of rt-PA in conjunction with PPV and gas tamponade has shown promising results for effective SMH displacement in various studies (5–7). Anti-VEGF, on the other hand, prevents rebleeding, inhibits neovascularization, and is typically applied postoperatively to inhibit the recurrence of the disease. Holz proposed the concurrent intravitreal injection of anti-VEGF drugs during surgical procedures involving rt-PA and expansile gas, demonstrating beneficial outcomes and favorable safety profiles (8). They believe that simultaneous intraoperative anti-VEGF may help limit disease progression and minimize the scaffold for scar formation. Ho further expanded this approach by combining intraoperative intravitreal anti-VEGF with PPV, subretinal air, and subretinal tPA injection, suggesting that the addition of anti-VEGF therapy could help maintain the post-displacement visual gain (9). The simultaneous application of rt-PA and anti-VEGF appears to be a promising treatment strategy, as the two agents act via different mechanisms and their co-application may improve treatment outcomes (10, 11). However, the optimal treatment approach for SMH caused by PCV remains uncertain. Further evidence is necessary to determine whether the co-application of subretinal rt-PA and intravitreal anti-VEGF yields better outcomes for PCV patients with SMH.

In the current study, we compared the treatment outcomes of PPV, rt-PA, and gas tamponade with or without simultaneous intravitreal anti-VEGF in PCV patients with SMH. Additionally, the study investigated whether the simultaneous application of anti-VEGF during surgery influences the subsequent need for anti-VEGF during an extended follow-up period.

## Methods

A retrospective analysis was performed on the medical records of patients with PCV and SMH who received pars plana vitrectomy and subretinal rt-PA injections at a tertiary hospital between January 2021 and March 2024.

The Institutional Review Board/Ethics Committee of the hospital approved this retrospective study (Approval No. S-K2033), and the study adhered to the Declaration of Helsinki. Written informed consent was obtained from all patients for both the procedure and inclusion in the study.

### Inclusion and exclusion criteria

The inclusion criteria were as follows: (1) a confirmed diagnosis of PCV with indocyanine green angiography (ICGA) or with the non-ICGA diagnostic criteria from the Asia-Pacific Ocular Imaging Society (APOIS) PCV Workgroup when ICGA was not available (12); (2) unscarred SMH was confirmed based on findings from fundus examination and optical coherence tomography (OCT); (3) patients who were treated for SMH using pars plana vitrectomy (PPV) combined with subretinal injection of recombinant tissue plasminogen activator (rt-PA); and (4) patients with a follow-up of at least 6 months after surgery. The exclusion criteria were as follows: (1) SMH secondary to other retinal or choroidal diseases such as AMD, retinal arterial macroaneurysms, or traumatic retinopathy and (2) patients whose medical records were incomplete or who did not complete follow-up. Prior to surgery, all patients received a comprehensive explanation of the procedural details, including the possibility of receiving additional anti-VEGF therapy. Patients expressed their understanding and signed the informed consent forms. Preoperative randomized allocation determined intraoperative anti-VEGF administration.

### Surgical procedure

The surgical procedure was conducted based on a report by Haupert et al. (13). All patients underwent a standard PPV procedure using a 25-gauge, three-port system under local retrobulbar anesthesia. Following the induction of posterior vitreous detachment and completion of core vitrectomy, the internal limiting membrane (ILM) was removed with the aid of indocyanine green (ICG) staining. Rt-PA (Actilyse^®^, Boehringer Ingelheim Pharma GmbH & Co. KG, Germany) was prepared by diluting it to a concentration of 0.25 mg/mL with a balanced salt solution. A 41-gauge subretinal infusion needle (MedOne, Sarasota, Florida, USA) was then used to inject ~0.1 mL of rt-PA into the subretinal space at a controlled pressure of 10 mmHg, facilitating the liquefaction of the SMH.

A fluid-air exchange was conducted, followed by the injection of 14% perfluoropropane (C3F8), to serve as a tamponade, facilitating the displacement of the SMH. At the conclusion of the procedure, intravitreal anti-VEGF was administered based on the randomization results. All intraoperative anti-VEGF administrations were delivered via the intravitreal route. Following surgery, patients were advised to maintain a facedown position for 5 days to optimize treatment outcomes.

### Postoperative treatment

Patients underwent monthly follow-ups for the initial 6-month period, followed by periodic evaluations at extended intervals ranging from 1 to 3 months. BCVA, intraocular pressure (IOP), FP, and OCT were evaluated at each visit. The need for re-treatment of surgical eyes was assessed through optical coherence tomography (OCT) imaging and fundus photography. Intravitreal anti-VEGF was administered on a pro re nata (PRN) basis in cases of subretinal fluid accumulation or recurrent SMH.

### Data collection

Patient baseline characteristics, including age, gender, the interval from SMH onset to surgery, prior treatments, pre-operative and postoperative logarithm of the minimum angle of resolution (logMAR) best corrected visual acuity (BCVA), and perioperative complications, were obtained from medical records. Swept-source optical coherence tomography (SS-OCT) imaging (VG200S, SVision Imaging, China; or DRI-1, Topcon, Japan) was used to evaluate SMH height, greatest linear dimension (GLD) of submacular hemorrhage, maximum pigment epithelial detachment (PED) height, and foveal involvement. GLD of submacular hemorrhage was defined as the maximal horizontal distance between points of neurosensory retinal protrusion. PED was evaluated using SS-OCT with a 12-line radial scan pattern (9 mm length, fovea-centered). The maximum PED height was defined as the greatest vertical distance between the apex of the detached retinal pigment epithelium and a reference line connecting its basal attachment points.

### Outcome measures

**Figure 1:**
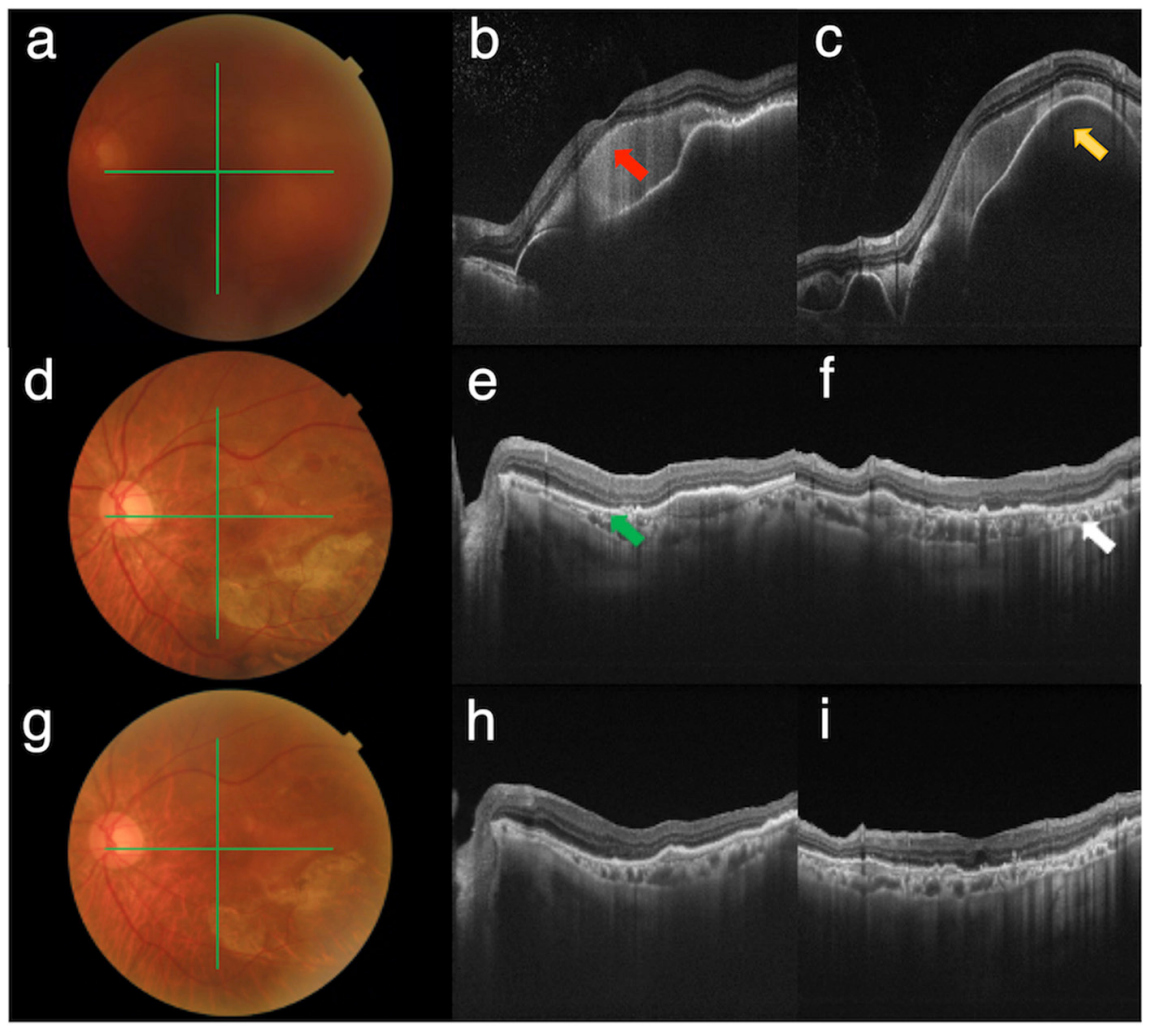
The left eye of a 67-year-old male with vitreous hemorrhage and submacular hemorrhage secondary to PCV. **(a)** Fundus photograph shows vitreous hemorrhage before surgery. **(b, c)** Swept-source optical coherence tomography (SS-OCT) shows massive SMH (red arrow) and large pigment epithelial detachment (PED) (yellow arrow). **(d)** Fundus photographs show complete absorption of SMH 2 months postoperatively. **(e, f)** SS-OCT shows complete absorption of SMH (green arrow) and flattening of PED. **(g)** Fundus photograph 8 months postoperatively shows stable fundus. **(h, i)** SS-OCT shows no recurrence of SMH.

The primary outcomes assessed were the comparative analysis of BCVA pre- and postoperatively, SMH absorption rate, and the number of anti-VEGF injections required following the surgical procedure. Vision levels were converted to logMAR values as follows: no light perception (NLP) was assigned a value of 2.90, light perception (LP) of 2.60, hand movement (HM) of 2.30, and finger counting (FC) of 1.85 (14). SMH absorption was categorized into three groups: “complete absorption,” “partial absorption,” and “no absorption.” “Complete absorption” indicated no presence of blood in the foveal region, as observed on postoperative fundus photography (FP) or OCT scans; “partial absorption” referred to a decrease in subfoveal blood with persistent blood or fibrosis in the fovea post-surgery; and “no absorption” indicated that there was no reduction in the quantity of subfoveal blood (Figure 1).

### Statistical analysis

Statistical analysis was carried out using Prism version 9. Variables that followed a normal distribution were expressed as mean ± standard deviation, while non-normally distributed variables were presented as median with range. The Kruskal-Wallis and Mann-Whitney *U*-tests were employed to compare continuous variables between groups. Changes in BCVA during the postoperative period were analyzed using repeated measures analysis of variance (ANOVA). The correlation between preoperative clinical parameters and final BCVA was examined using Spearman's analysis. A *p-value* of <0.05 was considered statistically significant.

## Results

### Patient characteristics

Tables 1, 2 summarize the characteristics of the enrolled patients. The study included 31 participants, with a mean age of 64.0 ± 8.3 years. The mean duration before surgery was 22.6 ± 12.8 days, and the mean follow-up was 13.2 ± 7.6 months postoperatively. A total of 15 patients (seven men and eight women) received intravitreal anti-VEGF during surgery (the anti-VEGF group), and 16 patients (seven men and nine women) did not receive simultaneous intravitreal anti-VEGF during surgery (the control group). Before the onset of SMH, 11 patients (68.8%) in the control group and 6 patients (40.0%) in the anti-VEGF group received intravitreal anti-VEGF injections (*p* = 0.156). Additionally, one patient in the anti-VEGF group received photodynamic therapy (PDT) before the onset of SMH. During surgery, 12 patients (80.0%) in the anti-VEGF group received intravitreal aflibercept injections, while the remaining three patients (20.0%) were treated with intravitreal conbercept injections.

**Table 1:** Clinical characteristics of PCV patients with SMH.

**Clinical characteristics**		**Anti-VEGF**	**Control**	***P***
Number		15	16	–
Age (year)		62.7 ± 7.2	65.2 ± 9.4	0.259
Male/Female		7/8	7/9	>0.999
Duration before surgery (day)		20.0 (23.0)	28.0 (55.0)	0.238
Foveal involvement		12 (80.0%)	13 (81.3%)	>0.999
Presence of vitreous hemorrhage		10 (66.7%)	11 (68.8%)	0.999
OCT	GLD (μm)	8,663 ± 2,776	9,094 ± 3,418	0.976
	SMH height (μm)	682 (1,716)	1,292 (1,372)	0.562
	PED height (μm)	666 (1,242)	1,164 (1,240)	0.175
	CMT (μm)	316 (728)	272 (1,285)	0.552
	CCT (μm)	301 (392)	257 (294)	0.483
Preoperative BCVA (logMAR)		1.52 (1.98)	2.30 (2.20)	0.207
Anti-VEGF before surgery		6 (40%)	11 (68.8%)	0.156
Number of anti-VEGF after surgery		1.0 (8.0)	1.0 (6.0)	0.679
Follow-up (month)		9.0 (28.0)	12.0 (20.0)	0.590
BCVA 6 months after surgery (logMAR)		1.19 ± 0.59	1.39 ± 0.74	0.499
BCVA at final visit (logMAR)		1.09 ± 0.54	1.46 ± 0.75	0.110
SMH absorption at 6 months after surgery	Complete	14 (93.3%)	14 (87.5%)	0.583
	Partial	1 (6.7%)	2 (12.5%)	
	No	0	0	

SMH, submacular hemorrhage; GLD, greatest linear dimension; PED, pigment epithelial detachment; CMT, central macular thickness; CCT, central choroid thickness; VEGF, vascular endothelial growth factor.

**Table 2:** Clinical data of PCV patients with SMH.

**Case No**.	**Age**	**follow-up (month)**	**Treatment before SMH**	**Duration before surgery (day)**	**Presence of VH**	**Simultaneous anti-VEGF**	**OCT (**μ**m)**		**BCVA (logMAR)**			**Number of anti-VEGF after surgery**	**Complications**
							**GLD**	**SMH height**	**Pre-op**	**6 months**	**Final**		
1	63	34	–	14	+	Ablifercept	NA	NA	1.00	1.00	1.00	0	
2	66	34	–	14	–	Ablifercept	4,961	647	0.92	0.60	0.92	4	
3	59	22	–	7	–	Ablifercept	11,937	2,226	1.85	1.30	0.70	1	
4	64	20	IVA^*^2	30	+	Conbercept	11,800	1,779	1.85	0.40	0.30	0	
5	67	20	IVA^*^11	21	+	Conbercept	10,311	1,636	2.90	2.00	1.22	8	
6	76	12	–	30	+	Ablifercept	5,135	510	1.52	1.85	1.70	5	
7	58	11	IVR^*^6, PDT	9	+	Ablifercept	1,2200	NA	1.00	1.00	0.82	0	Recurrent vitreous hemorrhage and secondary PPV
8	53	9	IVR^*^4	14	–	Conbercept	8,068	682	1.00	1.22	1.22	3	
9	67	8	–	20	+	Ablifercept	1,0853	672	1.70	1.00	1.00	3	
10	68	8	–	21	–	Ablifercept	6,422	548	1.00	0.10	0.10	4	
11	59	7	IVC^*^1	30	+	Ablifercept	NA	NA	2.30	1.30	1.30	1	Retinal detachment and secondary PPV+Silicon oil tamponade
12	59	7	–	10	+	Ablifercept	NA	NA	2.30	2.30	2.30	4	
13	47	6	IVC^*^3	7	+	Ablifercept	5,949	1,470	1.30	1.40	1.40	0	
14	63	6	–	30	–	Ablifercept	6,616	650	1.52	1.52	1.52	0	Macular hole and secondary PPV
15	71	6	–	30	+	Ablifercept	9,698	1,307	2.60	0.82	0.82	0	
16	65	26	IVA^*^8	30	+	–	NA	NA	1.30	1.10	1.22	3	
17	45	19	IVC^*^2	30	+	–	8,972	1,700	2.30	2.00	1.52	0	
18	51	17	IVA^*^1	46	+	–	4,887	1,160	2.30	2.00	2.30	0	Retinal detachment and secondary PPV+Silicon oil tamponade
19	67	14	IVR^*^1	30	+	–	5,538	1,292	1.75	0.70	0.30	0	
20	71	13	IVR^*^2	30	+	–	7,310	1,304	2.60	1.70	1.75	3	
21	72	13	IVR^*^1	10	–	–	9,374	939	1.52	0.70	1.00	1	
22	65	13	–	30	+	–	9,568	960	2.60	1.10	1.75	0	
23	61	12	IVR^*^1	7	+	–	NA	NA	2.30	1.15	1.75	5	
24	70	12	IVR^*^1	44	–	–	7,845	1821	2.30	2.30	2.30	0	
25	76	12	IVA^*^12	62	–	–	7,468	777	1.52	2.3	2.3	2	
26	82	10	IVA^*^10	26	+	–	NA	NA	1.05	1.22	1.00	6	
27	60	9	–	14	+	–	NA	NA	2.30	0.92	0.92	1	
28	67	9	–	14	–	–	16789	1633	0.40	0.22	0.40	2	
29	70	7	–	14	+	–	NA	NA	2.30	2.30	2.30	1	Recurrent SMH and secondary PPV
30	67	7	IVA^*^1	14	+	–	1,3509	1,406	2.60	2.30	2.30	0	
31	54	6	–	14	–	–	8,776	449	1.40	0.30	0.30	3	

SMH, submacular hemorrhage; VH, vitreous hemorrhage; OCT, optical coherence tomography; VEGF, vascular endothelial growth factor; BCVA, best-corrected visual acuity.

### Postoperative visual acuity and absorption of SMH

**Figure 2:**
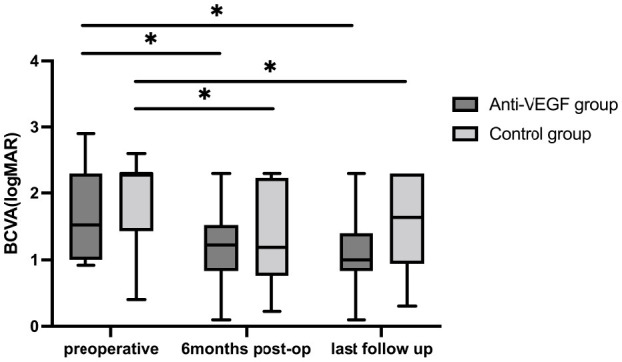
BCVA at baseline and follow-ups. Postoperative BCVAs were both significantly improved compared to baseline BCVA in the two groups. No statistical significance between the two groups in BCVA at each visit. *P < 0.05.

BCVA showed significant improvement in both groups at 6 months and the final postoperative follow-up. In the anti-VEGF group, BCVA improved to 1.19 ± 0.59 logMAR at 6 months (*p* = 0.035) and further to 1.09 ± 0.54 logMAR at the final follow-up (*p* = 0.024). Similarly, in the control group, BCVA improved to 1.39 ± 0.74 logMAR at 6 months (*p* = 0.014) and 1.46 ± 0.75 logMAR at the final follow-up (*p* = 0.024; Figure 2). There was no statistically significant difference in BCVA between the two groups at 6 months (*p* = 0.499) and at the final follow-up (*p* = 0.110). In the anti-VEGF group, eight patients (53.3%) experienced an improvement in BCVA, four (26.7%) maintained stable BCVA, and three (20.0%) exhibited a decline in BCVA at the final follow-up. In the control group, 11 patients (68.8%) showed improvement, four (25.0%) remained stable, and one (6.3%) showed worsening BCVA at the final follow-up. SMH was completely absorbed in 11 patients (73.3%) and partially absorbed in four patients (26.7%) in the anti-VEGF group at the last visit. In the control group, the SMH was completely absorbed in 13 patients (81.3%) and partially absorbed in three patients (18.8%) at the last visit. The average number of anti-VEGF injections administered during the follow-up period was 1.0 (8.0) in the anti-VEGF group and 1.0 (6.0) in the control group, with no statistically significant difference between groups (*p* = 0.679).

### Correlation analysis of preoperative parameters and final BCVA

Correlation analyses were performed between final BCVA and the following preoperative parameters: baseline BCVA, GLD, SMH height, PED height, and duration before surgery. The analysis revealed no significant correlations between final BCVA and preoperative GLD (*r* = −0.22, *P* = 0.306), SMH height (*r* = 0.10, *P* = 0.660), PED height (*r* = −0.12, *P* = 0.629), or surgical interval (*r* = 0.24, *P* = 0.189). In contrast, baseline BCVA demonstrated a statistically significant positive correlation with final BCVA (*r* = 0.485, *P* = 0.006), indicating that patients with better preoperative visual acuity achieved superior postoperative visual outcomes.

### Complications

In the anti-VEGF group, severe complications were observed in three eyes (20.0%), which included one case of retinal detachment, one case of macular hole, and one instance of recurrent vitreous hemorrhage. In the control group, retinal detachment and recurrent SMH were each observed in one eye (12.5%). All affected eyes subsequently underwent additional pars plana vitrectomy (PPV).

## Discussion

Our findings suggest that the addition of simultaneous intravitreal anti-VEGF does not provide a significant advantage in terms of visual improvement or reduced need for subsequent anti-VEGF injections when combined with PPV, subretinal rt-PA, and pneumatic tamponade for the treatment of SMH secondary to PCV. To the best of our knowledge, this is the first study to assess whether adding simultaneous intravitreal anti-VEGF provides an advantage over PPV, rt-PA, and gas tamponade alone in managing PCV-associated SMH.

Submacular hemorrhage, a serious vision-threatening complication of PCV, currently lacks a standardized management approach. Large-scale randomized trials are needed to establish evidence-based treatment guidelines (15). Rt-PA dissolves hemorrhages by degrading fibrin and coagulation factors (16). PPV combined with subretinal rt-PA and gas tamponade is effective for SMH (13, 17, 18), while anti-VEGF therapy, widely used for choroidal neovascularization (CNV), addresses SMH's underlying cause (19). However, the superiority of adding simultaneous intravitreal anti-VEGF to PPV, subretinal rt-PA, and gas tamponade remains unclear. In our study, visual acuity improvement and SMH displacement were achieved in both groups, with comparable outcomes in SMH absorption rates and subsequent anti-VEGF needs.

Guthoff et al. (20) demonstrated that the addition of intravitreal bevacizumab (IVB) enhances the displacement of submacular hemorrhages in AMD patients undergoing treatment with intravitreal rt-PA and gas alone. Our differing conclusion may result from procedural differences, as Guthoff's study used intravitreal rt-PA. Ma et al. (10) demonstrated promising vision and anatomical recovery with PPV, low-dose subretinal rt-PA, and intravitreal conbercept, though without a control group. Richmann et al. (21) reported no significant differences in visual acuity or structural outcomes between patients who underwent vitrectomy with subretinal rt-PA, whether or not intravitreal bevacizumab (IVB) was included in the procedure. Their findings align with ours, but our longer follow-up duration provides more robust data.

In SMH cases, thick subretinal blood clots may reduce the efficacy of intravitreal anti-VEGF, making subretinal injections a potential alternative. Delivering drugs directly to the subretinal space, the combination of rt-PA and anti-VEGF has shown efficacy in treating SMH secondary to AMD (5, 22, 23). However, controlled studies are needed to confirm whether subretinal anti-VEGF offers additional visual or structural benefits. Iglicki et al. (24) observed that subretinal administration of aflibercept resulted in greater visual improvement and a reduced need for anti-VEGF injections compared to intravitreal administration of aflibercept, with subsequent studies showing similar efficacy among aflibercept, ranibizumab, and bevacizumab (9). Further research with larger cohorts is required to establish optimal SMH management.

The most common complications of PPV, subretinal rt-PA, and gas tamponade include recurrent hemorrhage, retinal breaks, and retinal detachment (25–27). Recurrent vitreous or subretinal hemorrhage rates range from 16% to 73% (5, 28, 29), often necessitating repeat vitrectomies. In our study, each group reported a single case of retinal detachment and a single case of recurrent bleeding. All four affected eyes were treated with additional vitrectomy and silicon oil tamponade and remained stable after the removal of silicon oil during the follow-up period. One eye in the anti-VEGF group developed a macular hole 3 months post-surgery, and an additional PPV with C3F8 tamponade was performed. The macular hole closed after surgery, and the patient's vision remained stable during the follow-up period. The complication rates were similar between the two groups. We used a 41-gauge needle for subretinal injections to minimize retinal damage, though hemorrhage recurrence remains common due to PCV-associated large SMH. Hemorrhagic PED has been associated with recurrence (18). Kadonosono et al. (7) introduced a 47-gauge microneedle to reduce complications, yet macular holes and vitreous hemorrhage persisted. Substantial blood under the retina may cause high pressure, leading to breaks or VH. rt-PA's hemolytic action liquefies clots, potentially leaking into the vitreous and worsening VH. Caution is critical for massive SMH cases to mitigate severe complications.

The timing of surgery is a critical factor associated with visual prognosis. Research indicates that tissue damage begins within 24 h in cases of SMH and can lead to complete photoreceptor loss within 1 week (1). Early surgical intervention is recommended in previous research to achieve better anatomical and functional outcomes (24, 30). In our study, correlation analysis revealed no significant association between surgical interval and final BCVA. This finding aligns with the results reported by Juncal and Ogata (31, 32). The observed discrepancies across studies may stem from heterogeneity in patient populations, such as variations in baseline visual acuity, etiological profiles, or surgical indications. In our cohort, the minimum surgical interval was 7 days, with >50% of patients exceeding 14 days. As a tertiary referral center, patients typically undergo multi-tiered transfers before accessing our services, contributing to these prolonged wait times. The higher proportion of subjects with prolonged preoperative intervals likely hindered the establishment of a correlation between duration before surgery and final BCVA. Further exploration of the optimal time window for surgical intervention requires larger sample sizes and more rigorously designed prospective studies. Despite this relatively long waiting period, our study demonstrated that PPV, subretinal rt-PA, and gas tamponade remain effective in improving the vision of PCV patients with SMH. For example, in case 4 (anti-VEGF group) and case 19 (control group), both patients experienced symptoms for 30 days before surgery, with BCVA improving to 0.40 and 0.30 logMAR, respectively, at 6 months postoperatively. Both achieved complete SMH absorption.

Our study further confirms a significant correlation between baseline BCVA and final BCVA, aligning with established literature. Juncal et al. (31) specifically demonstrated that patients with a baseline BCVA >20/800 exhibited 5.90-fold higher odds of visual improvement post-surgery compared to the counting fingers/hand motion cohort. While superior baseline BCVA predicts better visual outcomes, surgical intervention remains beneficial even in patients with poor preoperative vision. Based on our clinical experience, individuals with symptom duration exceeding 2 weeks or severely compromised baseline vision may still derive meaningful functional gains from surgery.

The average number of post-surgery anti-VEGF injections was 1.0 (8.0) in the anti-VEGF group and 1.0 (6.0) in the control group. These figures are similar to those reported in other studies with a postoperative follow-up of 6 months (6, 33). Notably, our study indicated that simultaneous intravitreal anti-VEGF injection did not reduce the number of postoperative anti-VEGF injections required. The surgical procedure itself may be a more significant factor influencing postoperative treatment. In a study by Iglicki, AMD eyes with SMH were treated with either subretinal aflibercept or intravitreal aflibercept in combination with PPV, subretinal air, and subretinal tPA.

The study found that the subretinal aflibercept group required fewer anti-VEGF injections than the intravitreal group, averaging 6.2 ± 1.4 vs. 15.5 ± 1.9 injections over a 24-month period. Subretinal injection anti-VEGF delivers the drug molecules directly to the targeted neovascular membrane, and the higher concentration of subretinal anti-VEGF may more effectively suppress the neovascular membrane (24).

There are several limitations to the current study that warrant acknowledgment. The primary limitation is its retrospective design, which may introduce bias and limit the strength of the conclusions. Second, the study sample size was small, and the follow-up period was relatively short. Future studies with larger cohorts and longer follow-up periods are necessary to provide more robust evidence regarding the efficacy of simultaneous anti-VEGF administration during PPV surgery. Third, the disease duration before surgery in our cohort was longer compared to other studies. Additional studies that report treatment outcomes in patients with a disease duration of less than 14 days are needed to determine the impact of this treatment strategy on PCV patients with SMH.

## Conclusion

In conclusion, our study is the first to compare the efficacy of simultaneous intravitreal anti-VEGF administration within the context of PPV, subretinal rt-PA, and pneumatic tamponade against the same procedure without intravitreal anti-VEGF in PCV patients with SMH. Our findings indicate that the addition of simultaneous intravitreal anti-VEGF was not superior to the procedure without it in terms of visual gain and postoperative anti-VEGF usage. Both treatment strategies resulted in improved visual acuity. Further prospective, randomized controlled trials are warranted to establish the optimal treatment strategy for PCV patients with SMH.

## Data Availability

The raw data supporting the conclusions of this article will be made available by the authors, without undue reservation.
